# Hospital Meetings

**Published:** 1896-08-01

**Authors:** 


### HOSPITAL MEETINGS.

### THE NEW ROYAL HALIFAX INFIRMARY.

On July 25th T.R.H. the Duke and Duchess of York visited Halifax for the purpose of opening the new building which is to be known as the Royal Halifax Infirmary. The building which it is to replace was erected in 1838, and although several times enlarged, has long been found inadequate for the wants of the district. In 1889 it become obvious that something must be done to increase the accommodation, which was a matter of great difficulty on the old site. It was, therefore, decided to purchase a new and more extensive site on which a hospital containing between 300 and 400 beds could ultimately be erected, and to obtain complete plans for the whole before commencing the buildings immediately necessary.

A site of thirteen acres in the most healthy part of the town was obtained, and on it the new buildings have been erected, which it is hopod will be occupied before the end of the year.

The chief feature in the general arrangement of the infirmary is that the whole of that portion which is to be used by the patients is only one storey high, and that the wards, operating-room, administrative offices, dining-rooms, kitchens, &c., together with the surgery, dispensary, and casualty department, are on one floor.

Each pavilion consists of a ward containing twenty beds, and a small ward with two beds, together with a nurses' duty-room and a scullery. There is also a linen-room, a so-called splint-room, intended for bandages, dressings, splints, and all the lotions, ointments, and tests in daily use, and a room for patients' clothes. Cut off by a ventilating passage, there is a nurses' w.c., a housemaids' closet, and a small coal-store. At the further end of the large ward there are a bath-room and lavatory arrangements, all warmed by steam pipes, and cut off from the ward by a transverse ventilating passage; and in the case of those wards which open to the south a "sun-room," with large oriel window is placed between the bath-room on the one side and the sanitary arrangements on the other, and so separated from the ward by the ventilating passage that talking in the sun-room will not disturb the patients in the ward.

Each pavilion is, in fact, a small hospital, having its own stores and, as far as possible, working independently. The different parts of the infirmary are all connected together by telephone, the wires all going to an "exchange" in the porter's lodge. The corridors are heated by steam radiators. The main source of heat for the wards is by open fireplaces, but in each ward there are upright inlet ventilators entering direct from the open air, and containing steam heaters of sufficient capacity to warm the entering air. These ventilators are certainly not things of beauty, but they promise to be effectual. Each nurse's duty-room is provided with steam arrangements for warming food, &c. The whole of the buildings are lighted by electricity, produced on the spot by an engine of 52-horse power. Behind the kitchen are extensive washhouses and laundries, with the boiler-house, disinfector, and rooms for the engines, dynamos, and electrical storage.

The whole of the wards and corridors stand on arches clear of the ground, so that they are surrounded by air on every side.

The large wards are 88 feet long and 28 feet wide, and 17 feet high to the centre of the coved ceiling, there being thus nearly 9 feet of wall space per bed.

Four of the pavilions have out-floors, the other two being laid with "Terazzo," as also is the operating-room. The walls of the wards are of Keen's cement covered with four coats of Gay's enamel paint, and presenting a very hard and smooth surface. All angles are rounded. The windows are of plate glass, which is used for the sake of warmth, the lower third falling inwards when required, the upper being divided into louvres which open by a screw arrangement, and the middle third being fixed.

There are three ventilators in the ceiling of each ward, and the area of the inlets and outlets has been so calculated that it is hoped that even if from stress of weather it were necessary to close all the windows the wards would be efficiently ventilated. The operating theatre is a light airy room, the fittings of which are to be provided by the generosity of Mr. Hodgson Wright, the senior surgeon. Both it and the subsidiary rooms are heated by steam.

The accommodation for the nurses is very good, although we notice that separate bed-rooms do not seem to be provided for all the probationers. The nurses' sitting-rooms and library are extremely pleasant apartments.

The whole of the various administrative blocks and four of the ward pavilions are practically complete, and the other two pavilions are making rapid progress. The accommodation thus provided will be for about 140 beds. So far as it goes everything seems to be admirably arranged, but there is one blot upon the plan to which we feel bound to draw attention. No provision whatever seems to have been made for the isolation of such infectious diseases as may from time to time arise among the patients. Although various suggestions have been discussed nothing seems to have been decided. We would, however, strongly advise the committee not to bring these handsome buildings into use without making proper provision for the isolation of infectious cases. As the buildings stand at present the defect is too obvious to need further comment.

The proceedings on the occasion of the Royal visit were carried out punctually and satisfactorily. The Mayor (Mr. G. H. Smith) entertained the Royal party at a luncheon, to which a large number of the leading inhabitants were invited.

Sir Savile Crossley, the president, received the Duke and Duchess at the infirmary, and, after prayer by the Bishop of Wakefield, the Duke of York declared the new infirmary open, and announced that the Queen had given her permission that this infirmary in the future should be known as the Royal Halifax Infirmary.

The Treasurer (Mr. Whitley) then announced that he had received special donations, amounting to more than £14,000, for the reduction of the deficiency of about £20,000 which existed in the building fund. Among those presented to their Royal Highnesses, the medical staff was represented by Dr. Solomon Smith, consulting surgeon, and Mr. Hodgson Wright, the senior surgeon.

